# Facile Immunoassay Constructed by Gold Nanostar-Labeled Rabbit-AFP Antibody and Gold Nanoparticle-Conjugated Goat Anti-Rabbit IgG

**DOI:** 10.3390/nano15080612

**Published:** 2025-04-16

**Authors:** Kang Yang, Fang Yang, Xiaoling Lu, Hao Li, Zeng Yang, Qi Yin, Lin Zhang, You Long, Chao Shen, Liya Chen, Bo Yao, Chenghong Huang

**Affiliations:** School of Chemistry and Chemical Engineering, Chongqing University of Science & Technology, Chongqing 401331, China

**Keywords:** GNSs, AuNPs, immunoassay, AFP

## Abstract

Simple and accurate analysis of cancer-related biomarkers is very important for disease screening and auxiliary diagnosis. This study proposed a facile immunoassay that used gold nanostar-labeled rabbit anti-AFP as a capture antibody and gold nanoparticle-conjugated goat anti-rabbit IgG as an enhance antibody for the construction of a detection strategy for AFP analysis. Investigations indicated that the 50 nm diameter GNS-labeled capture antibody can specifically catch AFPs by direct detection profile or by further signal amplification through AuNP-tagged enhance antibody combination. Results showed that the developed method holds 8.6 ng/mL sensitivity, 20.0–110.0 ng/mL detection range, acceptable precision and fine accuracy, as well as favorable specificity. Results of application to real serum determination by the proposed method are highly related to those of the ECLIA method (correlation coefficient is 0.931). The proposed method has simple-operation merit and is very suitable for clinical screening of large-scale serum samples of cancers.

## 1. Introduction

Cancer is an important disease that endangers human health [1]. With the rapid increase in morbidity and mortality, early diagnosis and treatment of cancer are the keys to improving patients’ survival rate [2]. On the other hand, the levels of cancer biomarkers in serum are highly associated with its stages; thus, sensitive determination and accurate analysis of cancer biomarkers are very important to early screening and effective intervention [3]. Therefore, many efforts are contributed to developing novel methods to determine their concentrations in serum [4,5,6]. Generally speaking, measurement of biomarkers can be technically divided into label-free analysis and labeled analysis methods relying on the selected strategies. Label-free analysis for biomarkers refers to a method of detecting and quantifying biomarkers without the need for fluorescent, radioactive or other external labeling techniques [7]. Instead, it relies on the intrinsic properties of the molecules (e.g., mass, charge or optical characteristics) for identification and measurement; thus, it usually demands professional equipment and complex operations, while labeled analysis [8] refers to a method where specific biomarkers are detected, quantified or tracked using labeled molecules. These labels allow researchers to identify and measure biomarkers with high sensitivity and specificity. Currently, novel microsensors and nanosensors developed based on unique optical, electrical and magnetic materials with chemical principles are receiving increasing attention [9]. They often adopt non-covalent interactions involved in color change or pattern recognition for biomarkers and are known for their lack of accuracy [10], while immunoassay techniques based on antibody–antigen interaction behaving with distinctive specificity receive significant emphasis [11,12,13,14,15]. Therefore, the development of a new immunoassay with the merits of simplicity, applicability and accuracy is very important for the assisted diagnosis of cancer.

Recently, gold nanomaterials, generating new shape-dependent properties, appear to be an effective strategy for tuning the optical properties [16] of plasmonic metal in various geometries with different morphology and surface structure [17], and more attention needs to be paid as their specific atomic density, electronic structure and chemical reactivity may greatly enhance the performance of the sensor [18]. Among the most typical nanomaterials, gold nanostars (GNSs) have been considered an ideal photothermal conversion agent due to their facile large-scale preparation, tunable plasmon band, large absorption cross-section and high photothermal conversion efficiency [19]. Lately, the feasibility of single GNS as plasmonic transducers [20] for the construction of bio-SA assays based on the polarization response of individual nanostars to molecular binding has been proven. Wang [21] went a step further and developed a highly sensitive SERS-based sandwich immunoassay utilizing densely packed self-assembled substrates and labeled immune nanostar aggregates. What is more important for GNSs is that they are more conducive to biomolecular binding in dimensional structure compared to plate gold nanoparticles (AuNPs) or gold nanorods (GNRs). Hence, it is expected to develop high-performance biomarker immunosensors based on gold GNSs.

**Scheme 1:**
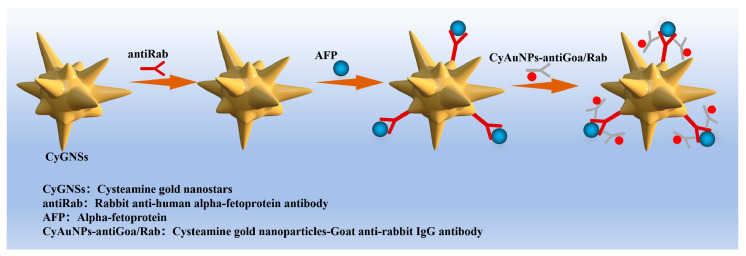
Illustration of immunoassay strategy constructed by gold nanostar-labeled rabbit anti-AFP IgG as a capture probe and gold nanoparticle-conjugated goat anti-rabbit IgG as an enhancer for AFP detection.

Alpha-fetoprotein (AFP) is an important biomarker for detecting primary liver carcinoma and for postoperative assessment [22]. Conditions will clinically be identified as (1) abnormal when AFP content is higher than 20.0 ng/mL, (2) suffering from cancer when 200.0 ng/mL AFP content lasts for 8 weeks, or (3) 400.00 ng/mL AFP concentration is sustained for 4 weeks. Cancer screening by tumor markers without injury is of very practical significance for health monitoring. This requires developing a simple operation and accurate measurement of AFPs for clinical application. This study aims to establish a simple immunoassay based on GNS-labeled rabbit anti-AFP IgG as a capture probe and gold nanoparticle-conjugated goat anti-rabbit IgG as an enhancer for AFP detection. The detailed construction strategy is illustrated in Scheme 1.

## 2. Experimental Section

### 2.1. Reagent and Apparatus

Tetrachlororiform acid (HAuCl_4_), cysteine, fetal bovine serum (FBS), hexadecyl trimethyl ammonium bromide (CTAB), 1-(3-Dimethylaminopropyl)-3-ethylcarbodiimide (EDC) and N-Hydroxysuccinimide (NHS) were collectively purchased from Aladdin reagents and used without further purification, Shanghai, China. Fetoprotein (AFP), rabbit anti-AFP antibody and goat anti-rabbit IgG were entirely gained from Huayang Zhenglong biochemical reagent Inc. Chengdu, China. Ascorbic acid (AA), absolute ethyl alcohol and sodium hydroxide (NaOH) were all bought from Chuandong chemical Inc. Chongqing, China. Serum samples were collected and stored in our laboratory. UV–visible spectrophotometry (UV-2600, Shimadzu, Japan), laser granulometry (Mastersizer 3000E, Liaoning, China), scanning electron microscopy (SEM, JSM-7800, Tokyo, Japan) and high-resolution transmission microscopy (HRTEM, JEM-2100F, Tokyo, Japan) were performed in CQUST. Deionized (DO) water was supplied in our laboratory.

### 2.2. GNSs’ Synthesis [23]

Firstly, 60 µL HAuCl_4_ (100.0 mM) was carefully added to 19.5 mL deionized water at room temperature, then 150.0 µL seed solution, 100 µL AgNO_3_ (3.0 mM) and 100 µL AA (100.0 mM) were successively added into the above solution and stirred for 10 min. The final product recovered by centrifugation was used for UV and TEM characterization.

### 2.3. Gold Nanoparticles’ Synthesis

The synthesis of citrate-capped AuNPs was referred to in the reported method [24]. The extinction spectra of the synthesized AuNPs were recorded by a double-beam UV-vis spectrophotometer. A total of 5.0 mL of as-prepared AuNPs were placed in a cuvette for UV-vis measurement. The scanning range was 400–1000^−1^. The size and morphology of AuNPs were simultaneously delivered for SEM observation. The operating voltage is 75 keV. Digital images of AuNPs with high resolution were collected.

### 2.4. Labeling of Antibodies

*Labeling of capture antibody:* A total of 0.6 mL (1 mM) of cysteine solution was added into 12.0 mL (optical density value, OD = 0.3) of GNSs solution to react for 1 h at 50 °C for the preparation of cystine-immobilized GNSs (CyGNSs). A total of 3.0 mL of rabbit anti-AFP was added into a 4.5 mL EDC/NHS (mole ratio = 3:1) mixture solution. The activated rabbit anti-AFP solution was then interfused into 18.0 mL of the aforementioned CyGNSs solution for 10 min. The capture probe of GNS-labeled rabbit anti-AFP was successfully prepared after 10,000 rpm centrifugation and precipitation resuspension in addition to final BSA blocking.

*Labeling of enhance antibody:* A total of 0.3 mL (50 μM) of cysteine solution was added into 12.0 mL (OD = 0.3) of AuNPs solution to react for 1 h at 50 °C for the preparation of cystine-immobilized cysteine-grafted AuNPs (CyAuNPs). A total of 10.0 mL of EDC/NHS (mole ratio = 3:1) activated goat anti-rabbit IgG was mingled with 12.0 mL of CyAuNPs solution to react for 10 min. Thereafter the enhancing probe was obtained after 13,000 rpm centrifugation, and precipitates were again resuspended in PBS buffer after 1.0 ug/mL BSA block action.

### 2.5. Combination of Antibody and Antigen

Sequence-diluted AFP solutions were added into capture antibody solutions for 60 min incubation at room temperature. The capture antibody will specifically catch hold of the corresponding antigen to form antigen/antibody composites. The aforementioned mixture was delivered to 4000 rpm centrifugation. The precipitates were uniformly resuspended in PBS once again. Then, different concentrations of the enhance antibody were added to explore the maximum concentration proportion.

### 2.6. Sensitivity, Reproducibility, Accuracy and Specificity

The minimum detectable limit of the immunoassay was determined by adding three standard deviations to the mean OD value of three zero standard replicates and calculating the corresponding values derived from the standard curve. Reproducibility was determined by evaluating the coefficient of variation (CV%) by applying 3.125 ng/mL, 6.25 ng/mL, 12.5 ng/mL, 25.0 ng/mL, 50.0 ng/mL, 100.0 ng/mL, 200.0 ng/mL and 400.0 ng/mL to perform the aforementioned immunoassay three times and to calculate average values. Accuracy of the immunoassay was determined by recovery assessment. Calibrated AFP standard (SD) solutions were spiked into the serum of determined concentrations to appraise the analysis accuracy. Specificity was assessed by signal comparison of blank control, positive control (100.0 ng/mL AFP), Ca^2+^ (2.25 mM), Cu^2+^ (21.8µM), Fe^2+^ (7.52 mM), glucose (3.9 mM), K^+^ (3.5 mM), Mg^2+^ (1.12 µM), Na^+^ (135 mM), Zn^2+^ (62.0 µM) and PBS (0.1 M), and was performed with related substances and their mixtures.

### 2.7. Real Serum Application and Comparison of Methodology

In total, 15 serum samples from a healthy person or a person with primary carcinoma were stored at −30 °C until the time of analysis. Samples or appropriate diluted samples were added to the capture antibody solution to react for 30 min at room temperature; the sample measuring was performed as described in the abovementioned procedures. Each sample was measured at least twice, and the mean value was accordingly calculated by the calibration curve.

## 3. Results and Discussions

### 3.1. Synthesis and Characterization of Nanomaterials

**Figure 1:**
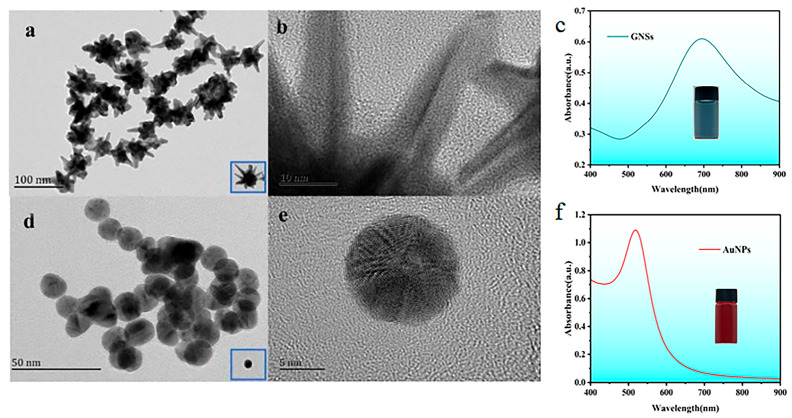
TEM images of GNSs (**a**), a detailed branch of an enlarged single GNS (**b**), AuNPs (**d**), an enlarged single AuNP (**e**) and their corresponding UV spectra (**c**,**f**). The inserts are real images of GNSs and AuNPs.

Gold nanostars (GNSs) are a type of anisotropic gold nanoparticles with multiple sharp branches [3], giving them unique plasmonic properties [25] that make them highly valuable in sensing applications [20,21]. Synthesized GNSs were spectroscopically located and investigated in a darkfield microscope equipped with a spectrometer coupled with a liquid-nitrogen-cooled, back-illuminated CCD camera (Figure 1a,b). The GNSs present multiple localized surface plasmon resonance (LSPR) [26] performances. A representative polarization-dependent scattering spectrum of a single GNS is shown in Figure 1c. Multipeak (Lorentzian) fits to these spectra revealed the presence of four resonances. GNSs may present more observable resonances; for consistency, we focused our single-particle investigations on GNSs whose spectra could be satisfactorily exclusively fitted with four resonances. The synthesized GNSs are provided with regular morphology as well as excellent dispersion. The diameter of AuNPs is approximately 5 nm (Figure 1d,e), displaying stronger UV light absorption at 520 nm (Figure 1f) and is basically consistent with the reported results [27].

### 3.2. Preparation of Capture Antibody and Enhance Antibody

**Figure 2:**
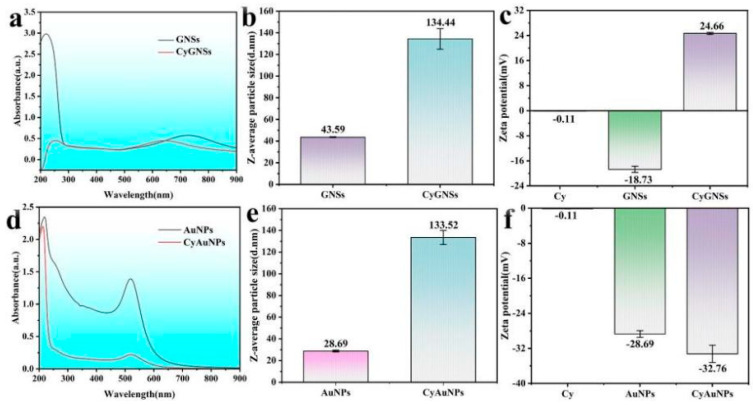
UV spectra (**a**,**d**), Z-average particle size distribution (**b**,**e**) and zeta potential changes (**c**,**f**) of GNSs and AuNPs after cys modification.

The as-prepared GNSs and AuNPs particles both exhibit aggregation properties due to relatively strong nanoscale effects. For subsequent antibody fixation, cysteine is used as an adaptor molecule to modify them for the acquisition of cysteine-modified GNSs (CyGNSs) and cysteine-modified AuNPs (CyAuNPs). There is a 20 nm blue-shift (Figure 2a) in the UV spectrum after GNSs are grafted by cysteine, as well as a larger size (Figure 2b) and a change in charge from negative to positive (Figure 2c). It is speculated that the increase in size is due to particle aggregation because of electrostatic attraction after grafting cysteine. While there exists an 18 nm red-shift in the UV spectrum after the AuNPs are modified by cysteine, simultaneously taking a stronger gathering phenomenon (Figure 2d) accompanied the average size change from 28.69 nm to 133.52 nm (Figure 2e) and bearing much stronger negative charges of altered charges from −28.69 mV to −32.76 mV (Figure 2f). It can be explained that after the sulfhydryl group of cysteine binds to gold nanomaterials, the remaining free amino and carboxyl groups mediated the accumulation due to electrostatic attraction.

**Figure 3:**
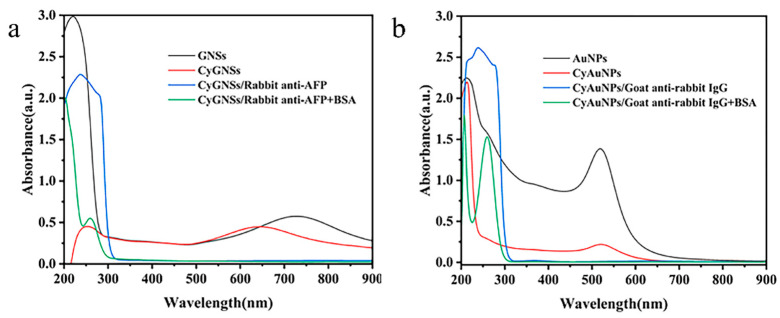
UV spectra of capture antibody preparation (**a**) and enhance antibody fabrication (**b**).

The aforementioned CyGNSs were first reacted with the EDC/NHS-activated rabbit anti-AFP antibody, and then the obtained product was designated as GNS-conjugated anti-AFP (Figure 3a). The latter was further accordingly called capture antibody. The likeness to AuNPs is AuNPs-labeled goat anti-rabbit IgG (Figure 3b) were named enhance antibody. They were both handled by 1.0 ug/mL BSA blocking treatment to prevent subsequent nonspecific adsorption of the irrelevant matter [28] after AFP addition and combination.

### 3.3. Establishment of Immunoassay

#### 3.3.1. AFP Direct Detection

**Figure 4:**
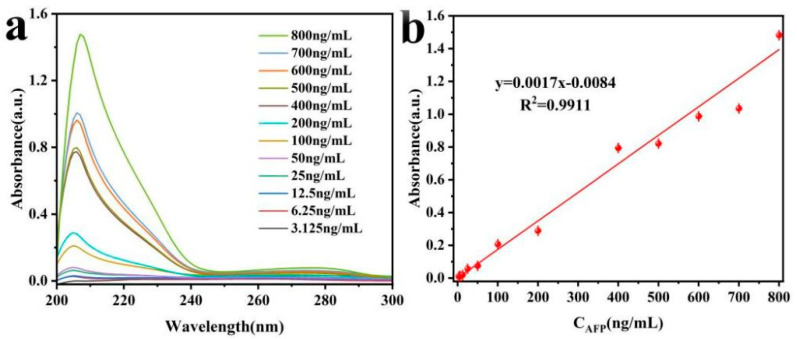
UV spectra of AFP direct detection via immobilization of 1.0 ug/mL capturing antibody and application of serially diluted AFP antigens (**a**) and corresponding fitting curve (**b**).

Preliminary experiments had testified that the 1.0 ug/mL capture antibody is the preferred option because of the effectiveness of the fixed antibody site on GNSs and the consideration of reagent cost. Two-fold diluted AFP SD solutions from an initial concentration of 800.0 ng/mL (800.0 ng/mL, 400.00 ng/mL, 200.0 ng/mL, 100.0 ng/mL, 50.0 ng/mL, 25.0 ng/mL, 12.5 ng/mL, 6.25 ng/mL, 3.125 ng/mL) were in proper sequence exposed to the capture antibody with a fixed concentration of 1.0 ug/mL to carry out antigen/antibody combination at room temperature for at least 30 min. Then the mixture was centrifuged at 5000 rpm, and the precipitates were resuspended in PBS buffer for UV determination. The results are shown in Figure 4. There was a quantitative relationship between AFP concentrations and OD values, with higher concentrations indicating higher OD values. The conclusion can be drawn that a lower S/N ratio is not suitable for quantitative detection as a result of inferior accuracy, especially for a high concentration of AFP. Therefore, direct detection can only be used for qualitative judgment but cannot be used for quantitative measurement.

#### 3.3.2. AFP Detection Through Signal Amplification by Enhance Antibody

**Figure 5:**
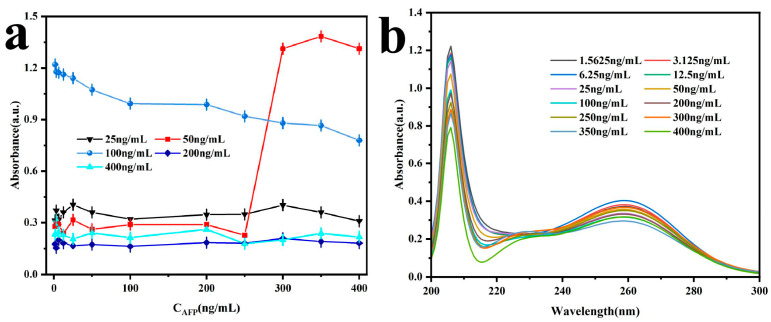
Concentration optimization of goat anti-rabbit IgG (**a**) and dose–response curve of AFP concentrations from 1.5625 ng/mL to 400.0 ng/mL (**b**).

An antibody, a typical Y-shaped glycoprotein, can specifically bind to a corresponding antigen through forces of hydrogen bonds, hydrophobic interactions, space matching, etc. [29]. This can gain high specificity, resulting in the goodness of fit between the paratope and the epitope, which is regarded as a feasibility measure foundation of immunoassays [30]. Experimental concentration to signal magnification by goat anti-rabbit IgG for AFP analysis was firstly optimized. As illustrated in Figure 5a, there was no obvious signal increase for the addition of less than 50.0 ng/mL AuNP-conjugated goat anti-rabbit IgG, while there was a notable signal augment when its concentrations were higher than that. Afterwards, by immobilization of AuNP-conjugated goat anti-rabbit IgG of 50.00 ng/mL and the application of different AFP antigens for immunoassay, the corresponding OD values were plotted and are indicated in Figure 5b. It can be seen that signal response is directly proportional to AFP concentration. The OD value from the blank control is 0.02 ± 0.01, but 0.08 ± 0.01 for the cut-off positive control (25.0 ng/mL), suggesting that the sensitivity was enough and the judge for normal or abnormal screening is enough. Since the AFP has at least three antigenic epitopes [28], this offered more rabbit anti-AFP antibody combination sites. It might be speculated that more goat anti-rabbit IgG will combine onto rabbit anti-AFP antibody because the stereospace structure of GNSs is well suited for large amounts of molecules to occupy position.

### 3.4. Characterization of Analyze Performance

#### 3.4.1. Sensitivity and Detection Range

GNSs taking unique shape structures and strong optical properties [31] are ideal for designing optical response detection in the biological field, as they can be easily handled by surface modification to strengthen colloidal stability and biocompatibility [19]. This also provides a good material basis for the accurate analysis of the markers associated with the disease. In terms of size, the GNSs are about 50 nanometers, while the AuNPs are only about 5 nanometers, so the space gap formed between them is just enough to accommodate the smaller gold nanoparticles. On the other hand, the multiple epitopes of AFPs also provide the technical feasibility of a single GNS to continue to bind multiple anti-IgG molecules. Therefore, the designed immunoassay has a good sensitivity. With PBS as a blank and a signal-to-noise ratio of 3, by plotting and calculation, the sensitivity achieved was 8.6 ng/mL. Effective detection range is between 20.0 ng/mL and 110.0 ng/mL, which is by and large able to meet the needs of clinical testing requirements.

#### 3.4.2. Precision and Accuracy

**Figure 6:**
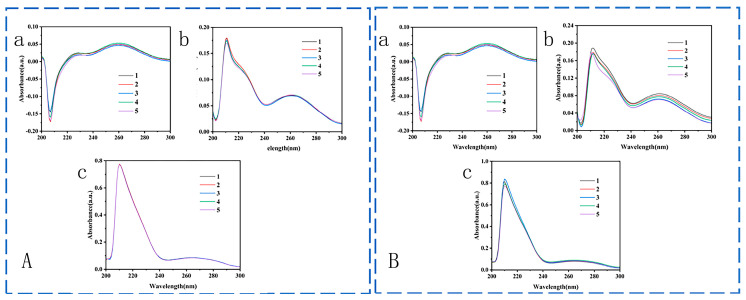
Group intra-repetitive UV-vis absorption spectra (**A**) and group-to-group repetitive UV-vis absorption spectra (**B**) for 25.0 ng/mL (a), 100.0 ng/mL (b) and 200.0 ng/mL (c) AFP concentrations. The numbers 1–5 denote five repeated measurements.

Precision was evaluated by group intra-repeatability and group-to-group repeatability. Group intra-repeatability was estimated by five times determination for the same AFP concentration, indicating the deviations are 1.87, 2.01 and 0.18 (Figure 6A) for 25.0 ng/mL, 100.0 ng/mL and 200.0 ng/mL, respectively, which is far lower than the requirements for the standard. Similarly, CV% values of group-to-group repeatability were 5.08%, 3.16% and 2.89% (Figure 6B).

**Figure 7:**
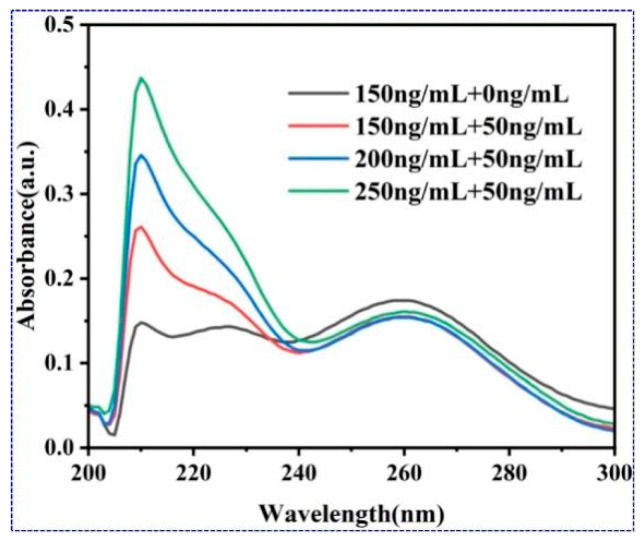
Determination of UV-vis absorption spectrum of recovery rates by immunoassay.

Accuracy was appraised by recovery estimation. 50.0 ng/mL AFP were orderly spiked into 150.0 ng/mL, 200.0 ng/mL and 250.0 ng/mL solution to prepare 200.0 ng/mL, 250.0 ng/mL and 300.0 ng/mL work concentrations. No AFP spiked sulotion was served as blank control. They were all tested by the developed immunoassay. The percentage of sample recovery rate was 103.6%, 103.2% and 103.1%, respectively, illustrating the proposed immunoassay holding a relatively acceptable accuracy (Figure 7).

#### 3.4.3. Specificity

**Figure 8:**
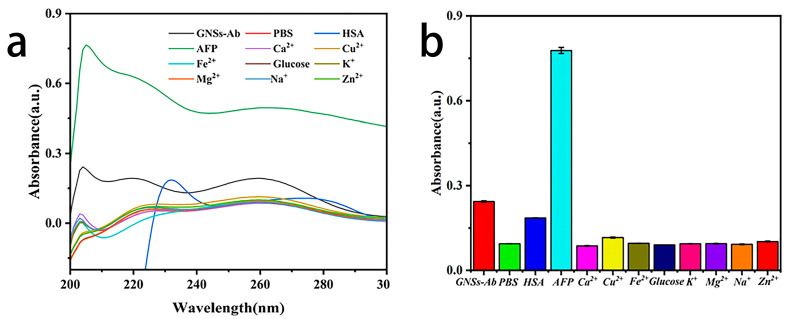
Determination of the specificity of AFP by UV-vis method: (**a**). UV absorption spectrum; (**b**) histogram of different substances’ absorption values.

Characterization of the specificity of the immunoassay was performed through evaluation of the relative signal values of 25.0 ng/mL AFP with the prepared interfering substances of PBS (0.1 M), Ca^2+^ (2.25 mM), Cu^2+^ (21.8 µM), Fe^2+^ (7.52 mM), glucose (3.9 mM), K^+^ (3.5 mM), Mg^2+^ (1.12 µM), Na^+^ (135 mM) and Zn^2+^ (62 µM), respectively. The UV value of 25.0 ng/mL AFP is much higher than that of the above interferent. It validated that the proposed method has acceptable specificity (Figure 8).

### 3.5. Methodological Comparison

**Figure 9:**
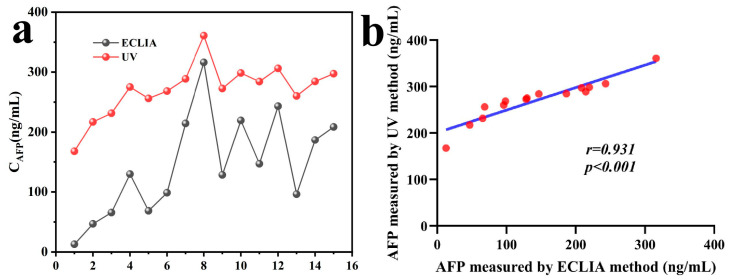
Detection of conventional ECLIA method and UV method with 15 serum samples: (**a**) serum concentration of the two methods; (**b**) correlation analysis.

The detection results of the 15 clinical serum samples using the immunoassay method established in this study were compared with those obtained by the commonly used clinical electrochemiluminescence immunoassay (ECLIA). As shown in Figure 9, the trends of the 15 serum samples detected by ECLIA and UV methods were consistent. It demonstrates a strong correlation (r = 0.931, *p* < 0.001) as analyzed by GraphPad Prism 10 CN. The two methods exhibited high consistency in the auxiliary diagnosis of HCC, indicating that this approach has a promising application potential in serum AFP measurement.

### 3.6. Future Work

This study leverages the spatial complementarity formed by the size differences between GNSs and AuNPs to establish an immunological method for detecting AFPs, using antibodies and conjugates for labeling. This method does not require cutting-edge equipment or specialized facilities, involves fewer steps and has low costs, while its sensitivity and accuracy fully meet clinical requirements, making it highly suitable for large-scale sample screening. The narrow detection range can be effectively solved by diluting the serum for further measurement. Future work should focus on further optimizing the synthesis of GNSs to achieve higher uniformity and longer branching structures. Additionally, improving antibody grafting efficiency to enhance binding activity is essential. Finally, standardizing reagents, operational procedures and analytical testing conditions is necessary to attain higher accuracy.

## 4. Conclusions

Simple and accurate analysis of cancer-related biomarkers is very important for disease screening and auxiliary diagnosis. This study proposed a facile immunoassay that used gold nanostar-labeled rabbit anti-AFP as a capture antibody and gold nanoparticle-conjugated goat anti-rabbit IgG as an enhance antibody for the construction of a detection strategy for AFP analysis. Investigations indicated that the 50 nm diameter GNS-labeled capture antibody can specifically catch AFPs by direct detection profile or further signal amplification through enhance antibody combination. Smaller-scale AuNPs are very easy to combine with larger-scale GNSs in space, leading to a single antibody-labeled GNS, and can combine multiple anti-antibody-conjugated AuNPs, which result in the developed method of good 8.6 ng/mL sensitivity, 20.0–110.0 ng/mL detection range, acceptable precision and accuracy, as well as favorable specificity. The proposed method still has simple-operation merit and is very suitable for clinical screening of samples.

## Data Availability

Data is contained within the article.
